# Scaffold Using Chitosan, Agarose, Cellulose, Dextran and Protein for Tissue Engineering—A Review

**DOI:** 10.3390/polym15061525

**Published:** 2023-03-19

**Authors:** Antony V. Samrot, Mahendran Sathiyasree, Sadiq Batcha Abdul Rahim, Robinson Emilin Renitta, Kasirajan Kasipandian, Sivasuriyan Krithika Shree, Deenadhayalan Rajalakshmi, Nagarajan Shobana, Shanmugaboopathi Dhiva, Sasi Abirami, Sridevi Visvanathan, Basanta Kumar Mohanty, Gokul Shankar Sabesan, Suresh V. Chinni

**Affiliations:** 1 School of Bioscience, Faculty of Medicine, Bioscience and Nursing, MAHSA University, Jalan SP2, Bandar Saujana Putra, Jenjarom 42610, Selangor, Malaysia; 2 Department of Biotechnology, School of Bio and Chemical Engineering Sathyabama Institute of Science and Technology, Chennai 600119, Tamil Nadu, India; 3 Faculty of Engineering, Built Environment and IT, MAHSA University, Jalan SP2, Bandar Saujana Putra, Jenjarom 42610, Selangor, Malaysia; 4 Department of Food Processing, Karunya Institute of Technology and Science, Coimbatore 641114, Tamil Nadu, India; 5 Department of Microbiology, Sree Narayana College, Alathur, Palakkad 678682, Kerala, India; 6 Department of Microbiology, Kamaraj College, Thoothukudi, Affiliated to Manonmaniam Sundaranar University, Thoothukudi 628003, Tamil Nadu, India; 7 Unit of Biochemistry, Faculty of Medicine, AIMST University, Semeling, Bedong 08100, Kedah Darul Aman, Malaysia; 8 Faculty of Medicine, Manipal University College Malaysia (MUCM), Jalan Padang Jambu, Bukit Baru 75150, Melaka, Malaysia; 9 Department of Biochemistry, Faculty of Medicine, Bioscience and Nursing, MAHSA University, Jalan SP2, Bandar Saujana Putra, Jenjarom 42610, Selangor, Malaysia; 10 Department of Periodontics, Saveetha Dental College and Hospitals, Saveetha Institute of Medical and Technical Sciences, Chennai 602105, Tamil Nadu, India

**Keywords:** nanotechnology, natural polymer, tissue engineering

## Abstract

Biological macromolecules like polysaccharides/proteins/glycoproteins have been widely used in the field of tissue engineering due to their ability to mimic the extracellular matrix of tissue. In addition to this, these macromolecules are found to have higher biocompatibility and no/lesser toxicity when compared to synthetic polymers. In recent years, scaffolds made up of proteins, polysaccharides, or glycoproteins have been highly used due to their tensile strength, biodegradability, and flexibility. This review is about the fabrication methods and applications of scaffolds made using various biological macromolecules, including polysaccharides like chitosan, agarose, cellulose, and dextran and proteins like soy proteins, zein proteins, etc. Biopolymer-based nanocomposite production and its application and limitations are also discussed in this review. This review also emphasizes the importance of using natural polymers rather than synthetic ones for developing scaffolds, as natural polymers have unique properties, like high biocompatibility, biodegradability, accessibility, stability, absence of toxicity, and low cost.

## 1. Introduction

**Figure 1:**
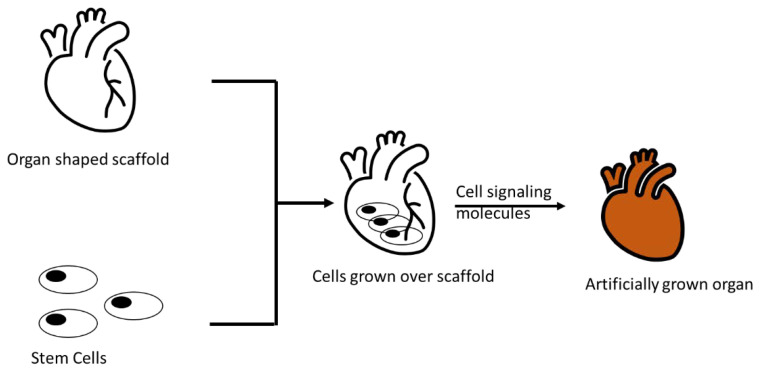
Tissue engineering process.

Nearly tens of thousands of individuals die each year as a result of the loss of organs or tissues or their dysfunction. Tissue engineering is one the powerful strategies for treating people who have lost or have failed organs or tissues [1]. Its triad relies on selecting the cells, the appropriate 3D scaffolds (mostly biopolymers based), and the appropriate chemical mediators required for tissue formation/regeneration (Figure 1) [2]. Tissue engineering is an in-vitro process for creating bioengineered tissues and an in-vivo process when implanting into a living system (mostly animals). In the multidisciplinary field of tissue regeneration, a lot of buzz is being generated, as it has proven to be a breakthrough therapeutic technique for solving the drawbacks of current artificial organs, as well as restoring severely damaged tissues or organs [3]. A scaffold is an artificial framework used for the formation of three–dimensional (3D) tissues, cell attachment and migration, and cell transport and retention, as well as the diffusion of essential nutrients and released products [4]. Scaffolds are expected to meet certain parameters, including excellent biodegradability, biocompatibility, a porous structure to facilitate cellular growth to help in tissue regeneration, and the controlled stimulation of the anticipated biological response to produce the desired product, which can be mostly tissue or organs [5,6] (Figure 1). These scaffolds can be altered to be formed at the nanoscale or microscale, which is expected to favor or regulate biological factors/growth factor releases [7,8].

Most scaffolds used in tissue engineering are made of porous materials which act or provide extracellular matrix or growth factors for cell growth; and they do have broader mechanical properties and are metabolically active [9,10]. Smaller pores enable tissue ingrowth, while larger pores (porous scaffolds) encourage cell seeding and migration. Many scaffold designs opt for high porosity materials with a porosity of above 90% because they allow for appropriate nutrient absorption during tissue culturing and offer enough surface area for interactions between cells and biomaterials [11]. After growing on the porous scaffold, cells/organs are transported from the laboratory to the real organism [12,13]. After the transfer of new tissue scaffolds to soft tissues in-vivo, do not necessarily need to be destroyed, whereas, for hard tissues, the scaffold materials do persist forever or for a certain duration. The scaffolds tend to stimulate the cells, and the tissue is eventually reconstructed by the messages from the scaffold [14,15]. The use of biomaterials in the tissue engineering field unlocks the innate regenerative potential of human tissues/organs, restoring them to their former state and restoring their function to normal [16]. The use of biomaterials, such as natural polymers, metals, ceramics, synthetic polymers, and their composites, in biomedical fields has been widespread for decades. Scaffolds are substances that are designed to encourage specific cellular interactions that aid in the development of functioning tissues [17,18,19,20]. The most significant aspect of any scaffold used in the tissue engineering field is that it must be biocompatible. An additional matrix must be layered after cells have adhered to the surface, which enables them to function normally, migrate through the scaffolds, and facilitate proliferation. A scaffold– or tissue–engineered construct will not trigger an inflammatory response that would limit healing or lead to rejection after implantation; rather, it will elicit a negligible immune response [17,19].

Biodegradable polymers used in tissue engineering are largely derived from medical implants and devices that are clinically proven. Among the natural macromolecules, agarose, collagen, alginate, derivatives of hyaluronic acid, fibrin glue, and chitosan have all been used as scaffolds [21,22,23]. The biologically active polypeptides called growth factors (GFs) control the repairing of tissue by interacting with specific receptors found on the cells. They play a major role in cell migration in the wound area, epithelialization, and angiogenesis, as well as stimulating the matrix formation and remodeling of the wound area. In addition to being short-lived and seeing rapid dissemination from the delivery site, as well as being relatively inexpensive, growth factors are often limited in their effectiveness [24,25].

In earlier days, potential biocompatible scaffold materials were extracted from donor tissue, where these biocompatible surfaces allow cells to divide and regenerate [26,27]. Biopolymers/biomaterials derived from bone, skin, blood vessels, and cartilage are regarded as suitable choices for tissue engineering. Nowadays, there are more biologically derived materials like polylactic acid (PLA), chitin, chitosan, collagens, polyether ether ketone (PEEK), polycaprolactone (PCL), fibrin, polyurethane, polyethylene (PE), polylactic–co–glycolic acid (PLGA), polyglycolic acid (PGA), and polyhydroxyalkanoates (PHAs) and their copolymers are used in tissue engineering and drug delivery [28,29,30,31,32]. These biomedical scaffolding requirements cannot be met by conventional single-component polymer materials and hence require a multicomponent polymer system [33]. When these polymers are made into nanocomposites, they can be used for imaging and targeting and show enormous potential in tissue engineering as they improve mechanical and functional properties and improve adhesion compared to conventional composites [34,35].

## 2. Polysaccharides in Tissue Engineering

Polysaccharides, including chitosan, alginate, dextran, and hyaluronic acid, have evolved lately in the field of scaffold research. They easily interact noncovalently to generate a loose viscoelastic gel in aqueous mediums and also have advantages like lower cost, simplicity in derivatization, biocompatibility, and biodegradability. Due to properties like these, polysaccharides are found to be comparable with extracellular matrix (ECM)-containing glycosaminoglycans, glycoproteins, and glycolipids [36]. The use of polysaccharides for various tissue engineering applications depends on the polysaccharide component as well as the biological aspects of the material’s interaction with the cells. Glycan units in polysaccharides have been associated with their capacity to provide biological signals [37]. Scaffolds must reflect both the ECM and the complexity of the target tissue through their composition and structure. The scaffold is commonly infused with angiogenic agents such as Vascular Endothelial Growth Factor (VEGF), Fibroblast Growth Factor (FGF) and Bone Morphogenetic Proteins (BMP2), Extracellular Matrix Proteins (ECM), and cell types that work well together [36,38].

### 2.1. Chitosan Based Scaffold and Its Synthesis

Chitosan is the second most abundant deacetylated form of linear biopolymer of chitin [29]. It has properties such as cell adhesion, cell survival, and cell interaction, and can be modified or used in its native form to create membranes, gels, sponges, scaffolds, and beads and can be used for a variety of applications like drug delivery and tissue engineering [39,40]. In tissue engineering, chitosan scaffolds are effective at avoiding fluid and nutrient loss due to their great water adsorption properties [41]. Chitosan-based scaffolds are made using a variety of fabrication techniques. Some of these are (i) freeze drying and subsequent gelation; (ii) salt leaching, and (iii) electrospinning [42]. Yang et al. [41] prepared a scaffold by a freeze-drying method in which the pre-gelling chitosan solution was mixed with dipotassium phosphate at 37 °C and pH was maintained at 7–7.2. The prepared chitosan scaffold showed 85 to 95% porosity. NIH/3T3 fibroblast cell lines grown over the chitosan scaffold were metabolically active and evenly distributed throughout the scaffold [41].

Chitosan incorporated with graphene oxide nanoparticles has shown good scaffolding properties, and high stimulation of cell growth as the hydrophilic characteristics of chitosan give it a favorable compatibility with regard to the formation of hydrogen bonds with graphene oxide nanoparticles. Chitosan-coated magnetic nanoparticles were used for seeding cells into the central portion of the 3D scaffolds, which facilitated cell invasion [43]. Poly(acrylic acid)- chitosan-TiO_2_ are reported to be suitable for bone based tissue engineering [44]. Chitosan coated with bio ceramic on zirconium is used in various therapeutic applications like surgical implants due to its better antimicrobial activity, low Young’s Modulus, and biocompatibility properties [45]. A 3D porous scaffold genipin crosslink matrix combined with gelatin, chitosan, and freeze-dried cellulose nanofibers was fabricated by Naseri et al. [46]. These scaffolds have nano/microscale pore wall roughness and linked pores with an average pore width of 75–200 μm, which makes them ideal for cell interactions in cartilage repair [46,47]. An ischemia-related disease can be treated successfully with the use of chitosan and hyaluronic acid-based nanocarriers. These nanocarriers are made using chitosan; they transport growth factors like vascular endothelial growth factor and platelet-derived growth factor [48].

Modified chitosan also plays an important role in bone tissue engineering. In a study, modified chitosan was freeze-dried at −20 °C, bearing imidazolyl groups that were covalently connected to the glucosamine nitrogen via methylene. It then underwent sterilization at a dose of 25 kGy, resulting in a soft, spongy, and hydrophilic final appearance. These were particularly appealing as a bone scaffold material promoting osteoblast cell adhesion and proliferation, as well as the in-vitro creation of a mineralized bone matrix. Additionally, investigations have demonstrated that in-vivo osteoconductivity is displayed by modified chitosan scaffolds in surgically induced bone lesions [39,49,50]. Due to its cationic characteristics, chitosan is thought to be able to combine with anionic macromolecules like glycosaminoglycans (GAGs) to modify the activity of cytokines and growth factors, and helps in the application of bone tissue engineering [42].

**Figure 2:**
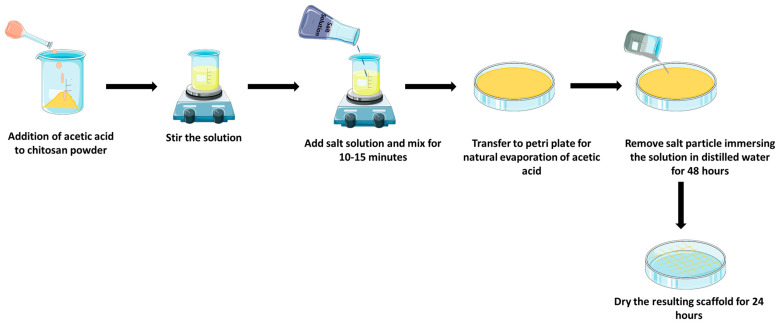
Preparation of chitosan scaffold using salt-leaching method.

**Figure 3:**
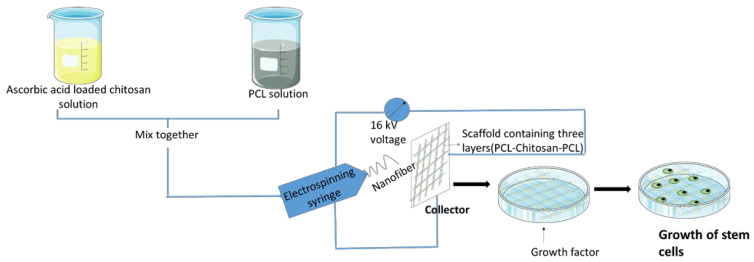
Preparation of chitosan scaffold using electrospinning method.

Chitosan-based scaffolds are generally made by a salt-leaching method or electrspinning method. In the salt-leaching method, water-soluble salt particles, such as sodium chloride, sodium acetate, etc., and a chitosan solution are mixed together for solidification, after which salt flakes are leached off (Figure 2), whereas, in the electrospinning method, charged chitosan threads are drawn into tiny chitosan nanofibers by electrostatic forces (Figure 3) [51,52,53]. The electrospinning method involves the usage of a polycaprolactone (PCL) solution along with the ascorbic acid loaded with a chitosan solution, which is prepared by mixing chitosan with acetic acid and ascorbic acid. A chitosan scaffold is made by electrospinning a PCL solution at first, and then the ascorbic acid-loaded chitosan solution is electrosprayed, and then finally electrospun using a PCL solution [54]. The addition of PCL to other polymers enhances their hydrophilicity, cell adhesion, stress crack resistance, and degradation rate. Chitosan with PCL is said to speed up hydrolytic degradation, increase wettability and permeability, and improves PCL cell recognition sites [55]. These chitosan scaffolds are found to be a prime candidate to be used as a sponge scaffold for orthopedic applications and regenerative bone therapy [53]. Good pore interconnectivity, better fiber thickness, a higher surface-to-volume ratio, and easier bioactive molecules (for incorporation) are considered to be the advantages of chitosan-based scaffolds. These are also utilized in regenerative medicine, where chitosan-based scaffolds make bioactive chemicals easily accessible to injured tissue, promoting wound-healing and regenerative effects with the least amount of morbidity and higher levels of biocompatibility [56,57]. In the case of bone tissue engineering, the disadvantages include poor mechanical properties, high rates of degradation, and low osteoinductivity [56].

### 2.2. Agarose Based Scaffold

Agarose is made up of linear, neutral polysaccharides, such as D–galactose and 3 ⮕ 6–anhydro–L–galactopyranose, and is commonly used in biological experiments like electrophoresis and bacterial cell culture due to their low interaction with biomolecules, physical and chemical stability, and thermoreversible gelation behavior. The biocompatibility of agarose makes it a promising biomaterial for tissue engineering and medication delivery. Biocompatibility and biodegradability characteristics are the most crucial elements in promoting tissue regeneration [58]. In order to create biological scaffolds that are adaptable to different kinds of soft tissues, agarose has also been mixed with other biomaterials. Agarose-based hydrogels are having good clinical applications for generation of human skin and other organs [59]. Agarose allows water to diffuse enough oxygen and vital nutrients for cells through its network. Agarose-based scaffolds for bone regeneration have recently been studied as the macromolecular structural characteristics of agarose and are similar to those of an extracellular matrix [60]. In addition, when subjected to static or dynamic loading, agarose behaves similarly to articular cartilage. Agarose and cartilage are both hydrating materials, so their hydraulic permeabilities are strain-dependent. This indicates that the hydrogel’s resistance to water transport increases as it is deformed and its pores collapse [61]. Agarose is an injectable polymer, which is eventually capable of being polymerizable in situ and is gaining popularity because of its reduced invasiveness during surgery, ability to be molded into the desired shape in situ, and potential for transporting cells and signaling molecules to the desired location [62]. Agarose scaffolds have also been found to improve the management of surgery and have increased cell phenotype consistency [61].

In a study, it was proposed that the regeneration of cartilage with a chondrocyte-encapsulated agarose scaffold maintains chondrocyte’s phenotypes and improves proteoglycan and glycosaminoglycan precipitation [62,63]. Despite the fact that agarose is an excellent cell carrier for tissue regeneration, few studies have explored its use in 3D tumor models. Its ability to provide a biocompatible 3D microenvironment that is favorable to tumor development is also investigated. Xu et al. [64] showed that examination of unique in-vivo ovarian cancer behaviors that are associated with the development of the disease, as well as testing for chemoresistance to anticancer medications, can be carried out by using agarose hydrogels/scaffolds. It was also demonstrated that the biomechanical properties of fibrin–agarose tissue-like hydrogels significantly improved than fibrin hydrogels, especially when chemical crosslinkers were utilized; it made it possible to successfully biofabricate a variety of biological alternatives with both ex-vivo and in-vivo results [65].

The most widely used method for the synthesis of agarose scaffold is the thermal crosslinking method. This method involves the usage of the gelation method, which can be carried out by microwaving. Varoni et al. [62] synthesized an agarose scaffold by a thermal crosslinking method, where 1.5% weight agarose was dissolved in an aqueous solvent by microwaving at temperatures varying between 60 °C and room temperature at a quenching rate of 30 °C/min. It showed improved cellular fibrous capsules and neutrophil development, for which the density was better than the commercially available gel materials, such as hyaluronic acid implants and collagen gel. The cellular activity and regeneration of the cells increased when the agarose was blended with other polymers. A combination of agarose and silk was used by Singh et al. [66] to rebuild cartilage. It was produced by mixing a dissolved agarose solution with a silk fibrin solution, followed by the lyophilization of the solution for 24 h. As a result of the silk/agarose scaffold, sulfated glycosaminoglycans (sGAG) and collagen deposition were more abundant, which implies the preservation of the chondrogenic phenotype. In blended hydrogels with collagen and fibronectin colocalization, the cartilage-specific marker genes aggrecan, *sox–9*, and collagen Type II were found to be upregulated to enhance the agarose microenvironment for chondrocyte culture. The interaction of collagen or fibronectin with chondrocytes and chondrocytes with living cartilage also improved matrix cohesion, which enhanced cartilage regeneration [59,67]. Yamada et al. [58] synthesized aldehyde-functionalized agarose hydrogels (CHO–agarose) by the oxidation of 2,2,6,6–(tetramethylpiperidin–1–yl)oxyl. Additionally, peptide–agarose microgel scaffolds were created by utilizing CHO-agarose and were successfully loaded with a cysteine residue present in peptide at the N terminus via thiazolidine polymerization. It has been found that a peptide–agarose microgel scaffold-based 3D cell culture system is a suitable biomaterial for tissue engineering due to the promotion of cell proliferation in a 3D environment [58].

**Figure 4:**
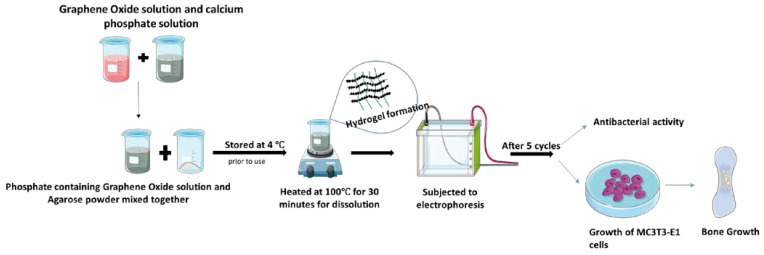
Synthesis of a graphene oxide/agarose biomaterial antibacterial activity and MC3T3–E1 cell attachment.

A nanocomposite consisting of agarose–gelatin–glass nanoparticles was depicted by Ali et al. [60]. It was synthesized by freeze gelation method, where agarose and gelatin were dissolved separately and stirred at 45 °C in distilled water. Then the glass nanoparticles were added to the gelatin mixture and mixed together with an agarose solution, which was followed by lyophilization in order to obtain a scaffold. These scaffolds could be used in the treatment of osteomyelitis and increase the hydroxyapatite layer present in body fluids, which promotes tissue regeneration and can be used as an excellent biomaterial [60]. Electrophoresis could also be employed for the synthesis of nanocomposites. A graphene oxide-containing agarose nanocomposite was prepared by mixing an agarose solution and a phosphate-containing graphene solution (a mixture of graphene oxide and calcium phosphate solution). This solution was further proceeded by electrophoresis for five cycles until layer formation occurred. This biomaterial exhibited promising results on antibacterial properties and excellent MC3T3–E1 cell adhesion (promotes high osteoblast differentiation), which can act as strong candidates for bone tissue engineering [68] (Figure 4). A modification of a chitosan–agarose–gelatin nanocomposite synthesized via gelation was carried out by using heparin nanoparticles by an ultrasonication method. This was found to have a high cytokine-loading capacity and prolonged the release of SDF–1 and BMP–2 cells, which makes it an excellent candidate for the repair and regeneration of bone [69]. Agarose-based scaffolds have strong physical crosslinking, thermoresponsiveness, and greater stability at lower concentrations as advantages. A drawback is that they are less soluble [70].

### 2.3. Cellulose-Based Scaffold

Cellulose is produced by joining D–glucose repeating units via glycosidic bonds. It is the most prevalent naturally occurring polymer on Earth, which is considered to be the primary component of plant cell walls. In addition to plants, some bacteria, including *Acetobacter xylinum*, *Pseudomonas* sp., *Agrobacterium* sp., etc., and fungi, tunicates, and green algae have/produce cellulose [71]. Using nanocellulose, a hybrid material that combines cellulose and nanomaterials, can have unique physicochemical characteristics. They, therefore, have exciting applications in biomedical sectors, including wound healing, drug transporters, 3D printing, bone tissue engineering, and medical implants [72,73,74]. Some of the other cellulose nanocomposites, such as nanowhiskers and nanofibers, are being applied as bio-nanocomposites to achieve antibacterial properties alongside matrices and fillers, such as metal oxide nanoparticles. Filler materials, such as clay, CNTs, and graphene oxide, can be added to the cellulosic matrix to demonstrate intracellular capabilities similar to tissue engineering and regenerative medicine [75]. Nanocellulose–based materials offer various benefits, such as biocompatibility, high optical transparency, water absorption, water retention, high performance, and outstanding mechanical qualities, which help it to act as a scaffold for tissue engineering. These also meets several essential requirements, including being biocompatible to mimic native tissue’s extracellular matrix (ECM) and supports in growth, proliferation, and the differentiation of cells [76]. Because of their adaptable surface chemistry and mechanical characteristics, cellulose scaffolds also act as a good material for 3D nerve cell growth and differentiation. Integrin-based attachment and cell–scaffold interactions in cellulose materials can be achieved by chemical modification and protein coating [77]. The benefits of this variety of scaffold include minimal manufacturing costs, biodegradability, nontoxicity, and biocompatibility. Despite its benefits, cell transport via cellulose-based scaffolds lacks cell recognition sites [78,79].

#### Synthesis of Cellulose Scaffold

**Figure 5:**
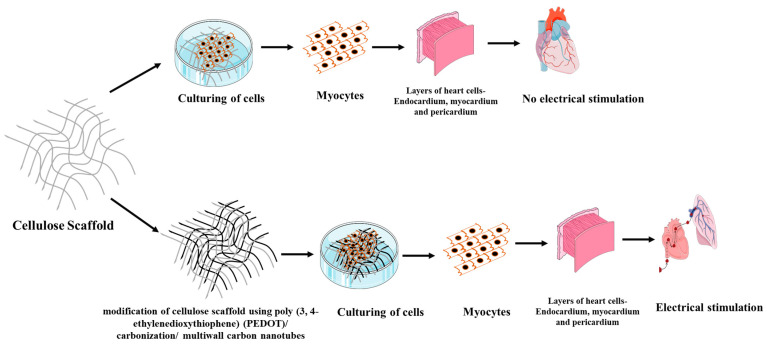
Modification of cellulose scaffold.

In nerve tissue engineering, electrical stimulation is a problem that is specific to a subset of cell types, such as neurons and myocytes. As a result, 3D nanostructured, electroactive biomaterials are needed. These requirements are achieved by the modification of cellulose scaffolds coated with conductive substances, like poly (3,4–ethylenedioxythiophene) (PEDOT), with the carbonization of multiwall carbon nanotubes [16,77,78,79,80] (Figure 5)**.** Novotna et al. [81] synthesized oxidized cellulose scaffolds by oxidizing it using organic solvents, such as Perfluorosol PFS–1 and nitrogen tetroxide. It was then followed by biofunctionalization using chitosan by exposing the scaffold to room temperature for a period of 2 h. Oxidized cellulose containing 2.1 wt% of –COOH is found to be relatively stable and biocompatible, and its allows the addition of other biomolecules such as chitosan. These may be appropriate for tissue engineering applications due to its relatively high stability under manipulation and exposure to cell culture conditions, as well as in the formation of bioartificial tissues due to their capacity for phenotypic maturation [81].

Similarly, 3D nanocomposite scaffolds made up of cellulose nanofibers blended with a chitosan/gelatin system were synthesized and used for cartilage regeneration. The synthesis of this nanocomposite was carried out using a freeze-drying technique, where the solution containing the nanofiber suspension and the gelatin/chitosan matrix were mixed together and dried at a temperature of −30 °C. This scaffold was found to have high porosity, moisture stability, and excellent cell–cell interactions. Thus, it can be used for ECM formation, which retains moisture in the interconnected pores, mimicking the natural cartilage in bone tissue engineering [47].

### 2.4. Alginate-Based Scaffold

Alginates are natural polymers found in brown seaweeds, such as *Ascophyllum*, *Durvillaea*, *Ecklonia*, *Laminaria*, *Lessonia*, *Macrocystis*, *Sargassum*, and *Turbinaria*. They are linear copolymers of D–mannuronic acid and L–guluronic acid that are connected by β(1⮕4) bonds [82]. These polymers, being naturally occurring multifunctional polymers with distinct physicochemical properties, have drawn increasing attention as desirable compounds in the biomedical and pharmaceutical industries over the past few decades due to their unique physicochemical properties and a broad range of biological activities [83]. The special ability of alginate to form a gel in aqueous environments led to this crosslinked hydrogel being used in tissue engineering, as well as in the delivery of bioactive molecules. These scaffolds are used to control the structure and function of engineered tissues, distribute cells to the desired spots, and facilitate the formation of new tissues because of the structural similarity of the alginate scaffold to that of ECM [84].

Alginate hydrogel is also an excellent choice for wound dressings due to its porosity, high water content, biocompatibility, and permeability to gases and water and its ability to enhance monocytes to produce high levels of cytokines like Interleukin–6 and tumor necrosis factor, which activate anti-inflammatory factors [85]. Additionally, antibiotic distribution in wound dressings is greatly influenced by the structural activity of the scaffold. The ability of the alginates to be changed or converted into biomaterials like hydrogels, wafers, fibers, biofilms, and foams helps to maintain a moist environment and to speed up the healing of wounds [86].

**Figure 6:**
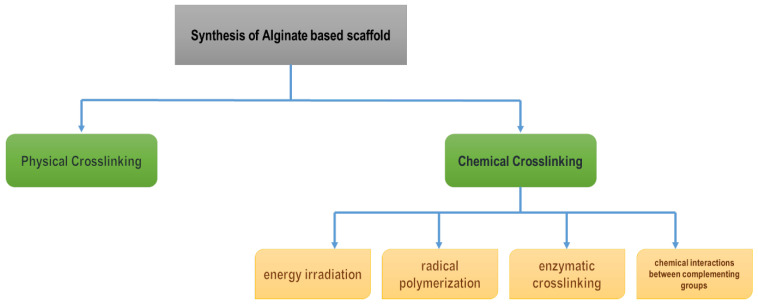
Synthesis of alginate-based scaffold.

Depending on the type of crosslinking and density, alginate has different mechanical properties [87]. Alginate scaffolds are produced using one of two crosslinking techniques: chemical or physical methods. The physical crosslinking of alginate hydrogels is the crosslinking of anionic polymer chains that can be connected by divalent cations such as calcium ions (Ca^2+^), strontium ions (Sr^2+^), and barium ions (Ba^2+^). The chemical method includes (i) energy irradiation; (ii) radical polymerization; (iii) enzymatic crosslinking, and (iv) chemical interactions between complementing groups (Figure 6) [87,88].

Alginate is often combined with other multifunctional materials, such as graphene oxide, polyvinyl alcohol, gelatin, hyaluronic acid, and skin fibroin, which can be used as ideal dressings for skin damage. These materials enhance various essential properties like cell adhesion ability, mechanical properties, and the hydrophilicity of the scaffolds [89]. In the field of regenerative therapeutics, these offer greater biocompatibility, ionic crosslinking, excellent vascularization, as well as faster wound healing capabilities that provide moist wound environments and have minimal inflammatory effects [70,90,91]. Despite their benefits, these molecules lack cellular interactions, which reduces their bioactive properties and their ability to support cell metabolism [91].

### 2.5. Dextran-Based Scaffold:

Dextran is an exopolysaccharide produced by lactic acid bacteria using sucrose as a substrate. This molecule is composed of a linear D–glucopyranose chain linked by *α* (1⮕6) bonds as the main chain with variable amounts of branching linkages, like *α*–(1⮕2), *α*–(1⮕3), and *α*–(1⮕4), with molecular weights around 40 kDa [92,93]. It can be modified with different functional groups to form spherical, tubular, and 3D networks because of its chemically reactive hydroxyl groups. Biodegradable dextran-based scaffolds can function as bioactive carriers for a variety of protein biomolecules for effective regulated release and tissue regeneration [94]. Dextran-derived hydrogels can also be used as bioartificial cardiac tissue matrix (BCT) for in-vitro cardiac tissue regeneration [95].

Dextran hydrogel scaffolds have been determined to be advantageous as scaffolds that can be used only for soft tissue engineering because they exhibit high resistance towards protein adsorption and cell adhesion, enabling the design of scaffolds with specific recognition sites. Lévesque et al. [96] depicted the synthesis of a crosslinked network of dextran hydrogels with radical methacrylate group polymerization. The immiscibility between the dextran matrix and poly(ethylene glycol) in aqueous solutions made macroporous, beaded-wall morphology scaffolds that can manage liquid–liquid phase separations and were found to increase cell penetration and nutrient diffusion [96]. They can act as bioactive carriers for numerous protein biomolecules that are inherently biodegradable but are said to be higher in cost and have less bioavailability [97,98].

## 3. Proteins Based Scaffolds

Proteins obtained from plants, such as zein, soy protein, and wheat gluten, are used in the field of tissue engineering as they have low immunogenicity when compared to animal proteins [99]. Further, they are more polar and have a lower molecular weight than animal proteins, making them naturally hydrophilic and effective cell attachments. The characteristics of plant tissues make them uniquely suited for use as scaffolds, with pre-existing vascular networks, including high surface areas, good mechanical properties, interconnected porosity, and excellent water absorption [100]. The fabrication of the scaffolds using plant protein can be carried out by using physical, chemical, crosslinking, and electrospun methods [99]. Zein is the main storage protein found in corn endosperm and accounts for 40–50% of the total endosperm proteins. Zein is biocompatible with human umbilical vein endothelial cells, mouse fibroblast cells, and human liver cells. Zein, therefore, has the potential to be used both as an engineered scaffold and a vehicle for medication delivery [100]. Scaffolds fabricated using soy protein (3% soy protein isolate crosslinked with transglutaminase) increased cell spreading, with the cells integrating into the scaffold within two weeks, demonstrating the ability of the porous scaffolds for tissue regeneration projects [99,100,101,102].

Proteins such as collagen, fibronectin, silk protein, elastin, and albumin, are also used in the synthesis of nanoparticles [103]. Almost all human and animal tissues contain collagen as their primary structural protein, a component of extracellular matrix (ECM) and tissues for their structural support and to maintain their biological integrity [104]. When used as a scaffold, collagen offers a wide range of benefits for cell-based tissue repair. A porous collagen scaffold made from pepsin-digested (e.g., telopeptide–free) bovine skin collagen was found to be biocompatible when implanted into healthy rat subcutaneous pockets than scaffolds made from acid-soluble bovine skin collagen [105,106]. Soy protein is known for its biocompatibility and stability and, hence, can be used in tissue engineering [107]. In bone tissue, collagen is the most abundantly found polymer. When collagen is incorporated into composites, more cell recognition sites are provided, as well as a faster rate of biomaterial degradation, ensuring rapid bone regeneration [108]. In a study, nano-HA (hydroxyapatite) crystals were positioned along the collagen molecules in the HA–Type I collagen nanocomposite. An HA–collagen composite was found to promote bone remodeling and has high osteoconductive activity. The highest concentration of rhBMP–2 (400 g/mL) reduced the length of time needed for bone union when the implants were grafted at weight-bearing sites [108,109].

Carbon nanoparticles that are made using proteins like hydroxyapatite/collagen (C)/poly(lactic–co–glycolic acid)/graphene oxide (nHAp/C/PLGA/GO) have greater mechanical strength and material longevity, which plays a main role in the design of tissue engineering scaffolds and helps in the proliferation of MC3T3–E1 cells aided by composite scaffolds [110]. In photodynamic therapy applications, high-density lipoprotein nanoparticles may be beneficial because they possess excellent tumor targeting and internalization capabilities. Albumin, the most abundant plasma protein, were employed to promote bone regeneration by releasing Bone Morphogenetic Protein–2 over time (BMP-2) [111]. In earlier studies, the nano–HA crystals were positioned along the collagen molecules in the HA–Type I collagen nanocomposite.

**Figure 7:**
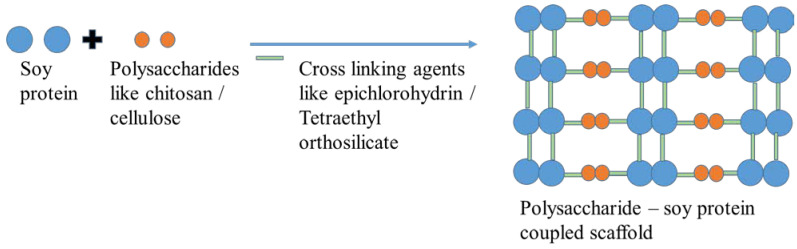
Synthesis of soya protein-based scaffold using polysaccharide.

The common method used for the production of protein-based scaffolds are electrospinning, freeze drying, solvent casting, sol-gel methods, etc. Silva et al. [112] produced porous scaffolds using chitosan and soy protein via a sol-gel and freeze-drying method. Tetraethyl orthosilicate is a linker that links chitosan and soy protein and enhances their mechanical stability, water uptake, porosity, etc. [113]. Zhao et al. [114] produced hydroxyethyl cellulose–soy protein using epichlorohydrin (ECH) as a crosslinking agent; the resultant copolymers showed good biodegradability and biocompatibility and allowed L929 fibroblast cells to adhere and grow well (Figure 7).

### Zein-Based Scaffold

**Figure 8:**
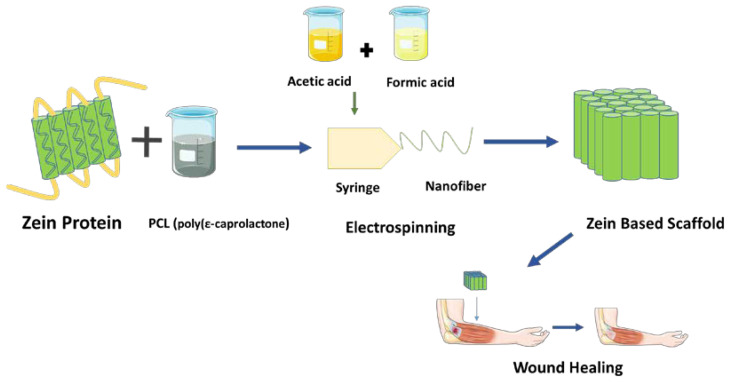
Synthesis of zein-based scaffold using PCL (poly(ε–caprolactone).

Zein, a type of alcohol-soluble prolamine that is found in maize endosperm, is composed mainly of three compounds: (i) *α*–zein, (ii) *β*–zein, and (iii) *γ*–zein. It was recognized as a structural protein for gluten-free systems due to its ability to create viscoelastic networks similar to gluten [115]. Plath et al. [116] depicted the synthesis of zein scaffolds with a weight percentage of 40 by blending it with PCL (poly(ε–caprolactone) via an electrospinning method and a binary solvent system consisting of acetic acid and formic acid. These scaffolds appeared to have a decreased microbial adhesion in tissues or biomaterial components during the repair phase of tissue regeneration, thus being a promising application for wound healing and skin repair properties. The antibacterial activity of the scaffold was exhibited by the cationic ion –NH_3_^+^ present in the scaffold provided by the binary solvent system [116] (Figure 8). In a study, it was discovered that all PCL/zein/gum arabica scaffolds had porosity levels higher than 77%, making them appropriate and advisable for cell infiltration. In addition, this composite scaffold displayed antibacterial characteristics as a result of the cyanogenic glycosides found in gum arabic, along with improved hydrophilicity. The hydrophilicity of a scaffold is important because it promotes cell viability and growth [117,118].

Similarly, a zein scaffold made using both higher concentrations (70–80%) and lower concentrations (70–80%) exhibited high compressive and tensile strength, a good bending modulus, and high elasticity. It showed an excellent repair efficacy in rabbits with bone defects of size 15 mm when compared to commercially available *β*–tricalcium phosphate [119]. Changing the blending ratio showed variable surface wettability, mechanical strength, fiber diameter, and in-vitro degradation capabilities, as well as cell adhesive properties in electrospun membranes. The application of zein protein improves electrospinnability and fiber tensile strength, while collagen promotes surface wettability, in–vitro degradability, and cell adhesion [120].

Zein scaffold blended with gelatin was synthesized via an electrospinning method. In this method, different concentrations of gelatin were obtained by using 1,1,1,3,3,3–hexafluoro–2–propanol, and both the zein and gelatin solution were electrospun in order to obtain a nanoporous scaffold. Better electrospinnability was achieved between zein and gelatin, which resulted in improved mechanical properties, hydrophilicity, and cell adhesiveness of the zein membranes [121]. In bone tissue engineering, zein-based scaffolds have a number of benefits, including the development of osteogenic properties and good mucoadhesive properties for drug delivery applications [122,123,124].

## 4. Metallic Nanoparticles for Tissue Engineering

Metal nanoparticles have been extensively used in tissue engineering. Several metal nanoparticles, such as gold, silver, iron, aluminum, nickel, copper, and zirconium, as well as magnetic nanoparticles, have been investigated for this purpose. Metallic nanoparticles are highly recommended for bone implants since they are well-established in the field, long-lasting, sturdy, and biocompatible [121]. Scaffolds made from silver have various advantages, such as high cell adhesion and spreading, high proliferation, osteoconductivity, capability with bridging oxygen molecules, improved osteogenic properties, good cytocompatibility, good mechanical strength, effective antibacterial activity, and also low toxicity [125]. Metallic nanoparticles are useful for bone tissue engineering because they activate osteoblasts, inhibit osteoclasts, provide mechanical strength and antibacterial action, and, in some circumstances, stimulate angiogenesis [126]. Furthermore, the magnetization of nanoparticles used in orthopedics repair scaffolds may be achieved via mechanostimulation. The nanomotion produced by the magnetic field on the scaffolds produces forces in the pH range, and cells respond to these mechanical stimuli by releasing ATP, contracting cytoplasmatic actin, and expressing FAK (Focal Adhesion Kinase), which is the source of chemical signals promoting cell growth and differentiation. In this case, mechanotransduction using magnetic scaffolds help in transmitting impulses deeper into the tissue [127]. Magnetic iron oxide nanoparticles are frequently used in the promotion of bone growth, drug loading, stem cell-based bone development, and scaffold-based bone formation by their magnetic stimulation [128,129,130,131,132,133,134]. In a study, magnetite nanoparticles were employed to build multilayered cell-sheet-like structures and also tubular structures. These functionalized magnetite nanoparticles were reported to boost tissue engineering procedures [135]. SPIONs (Superparamagnetic Iron Oxide Nanoparticles) have also been used as a contrasting agent in X-ray imaging and magnetic resonance imaging (MRI) [129,130]. It provided a better contrast to the background and showed the egg yolk’s distinct boundaries; it is reported to enhance the effect of X-rays. In a study, novel super paramagnetic iron oxide nanoparticles with monoclonal antibodies were developed and used for the detection of endothelial inflammation, which is a defining characteristic of many illnesses connected to endothelial dysfunction, including atherosclerosis, diabetes, and cancer metastasis [130]. Studies also suggest the usage of Superparamagnetic Iron Oxide Nanoparticles (SPIONs) and quantum dots in tracking cell biodistribution [33,136]. The super paramagnetic iron oxide nanoparticle (SPION)-labeled mesenchymal stem cell’s (MSC) tumor tropism and biodistribution in the orthotopic model of C6 glioblastoma in Wistar rats were evaluated. When SPION-labeled MSCs were administered intravenously in living organisms, the tagged cells accumulated inside the tumor site. This dramatically improved the tumor’s contrast on high-field magnetic resonance imaging [137].

## 5. Nanotechnology in Tissue Engineering

The increased surface area of nanoparticles can improve the bioactivity of scaffolds, and can be functionalized with proteins or other biopolymers to stimulate bone cell adhesion, proliferation, and differentiation [138]. Due to van der Waals forces, nanoparticles can form clusters, which can lead to the formation of uniformly dispersed scaffolds. Since bone cells have a rough surface and holes of roughly 2100 nm, they are claimed to react organically with nanostructured materials. Generally, nanoparticles such as gold, silver, magnetic, ceramic, bioresorbable nanoceramics, titanium dioxide, polymeric nanoparticles, etc., are used in the implantation of tissues and in the delivery of the growth factors required for tissue generation [111]. As nanomaterials can be incorporated into all proteins, such as fibronectin, collagen laminin, and vitronectin, that are responsible for osteoblast function (over conventional-sized materials), their use in bone regeneration has been urged on by the growth of cells and the adhesion of nanocomposites [139]. In addition to their chemical composition and similarity in structure to natural bone, they also possess unique functionalities (like a large surface area) and superior mechanical strength compared to their single-phase counterparts. On the other hand, nanoparticles that are functionalized with an appropriate biopolymer can simulate such roughness [140]. Nanoparticles with antibacterial and antifungal properties, such as zirconium, gold, titanium, and silver oxides, are useful in preventing infections caused by micro-organisms [141]. Carbon nanotubes (CNTs) are cylindrical carbon derivatives with a length/diameter ratio of 28,000,000/1, where single-wall CNTs and multiwall nanotubes CNTs are utilized to make scaffolds [142]. Graphene oxide-coated collagen scaffolds have shown improved cell proliferation, bioactivity, and differentiation both in-vivo and in-vitro with low GO inclusion [143]. The addition of a carbonyl group to a zinc oxide/carboxylated graphene oxide nanocomposite was studied, and it showed increased ALP activity, extracellular matrix mineralization, up-regulated osteogenic–related genes (OCN, ALP, and RUNX2) in MG63 osteoblast–like cells, and also antibacterial activity against *Streptococcus mutans* [16].

A number of other 2D nanomaterials exist, such as hexagonal boron nitride (hBN), black phosphorus, and graphitic (C_3_N_4_), and elemental monolayers, such as anti-monene, germanene, silicone, and arsenic [144]. The thinness of 2D nanomaterials also makes them ideal for a range of optical applications (such as imaging), as their thinness enables them to react swiftly to external stimuli [145,146,147].

**Figure 9:**
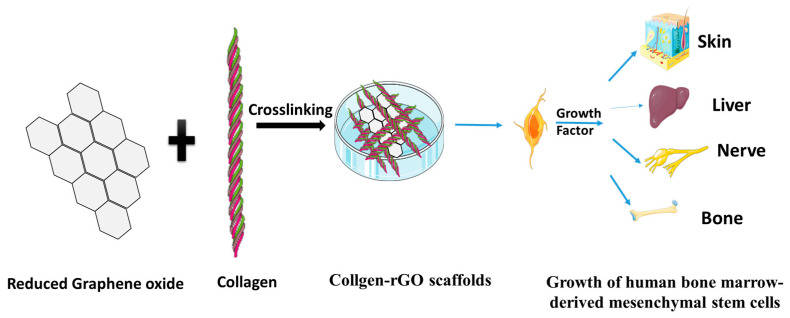
Collagen–rGO scaffold via crosslinking.

In cardiac tissue engineering, electrophysiological impulses are sent by the myocardium, making excellent conductivity one of the most crucial characteristics for biomaterials used to treat cardiac damage. Graphene oxide plays a great role in ECM hydrogel therapies for treating myocardial injury. Graphene hydrogels made from the coprecipitation of tricalcium silicate showed higher cell viability and proliferation of cardiomyocytes and fibroblasts through great adhesiveness, self-healing properties, and conductivity [148]. In addition, regarding hydrophobic contacts, electrostatic forces, and hydrogen bonding, GO can take up ECM proteins. When MSCs and GO were cocultured with ECM, improved cell adhesion and survival under ROS conditions were seen in-vitro [149]. A graphene oxide scaffold developed by the incorporation of silk fibroin had increased surface roughness and high protein adsorption, and high electrical conductivity, helping in the culturing of neurons [150]. It has also been evidenced that graphene improves mechanical strength and cytocompatibility. In addition, it accelerates hMSC adhesion, proliferation, and differentiation toward osteogenic cell destiny [145,146,147,148,149,150,151]. A graphene scaffold made by crosslinking collagen via freeze-dying showed better growth of mesenchymal stem cells [143,152] (Figure 9).

The modification of reduced graphene oxide with hydroxyapatite was found to enhance the regeneration of new bone formation. Similarly, reduced grapheme oxide functionalized with chitosan and silk fibrin showed improvements in the hydrophilicity and growth of cells such as G–292 cells [153,154,155]. TMDs are layered materials with a structure resembling that of grapheme that are made of transition metal atoms sandwiched between layers of chalcogen atoms [156]. TMDs, like molybdenum disulfide functionalized with chitosan, were found to cavity-up the joint in the condition of osteoarthritis and showed no sign of erosion in the cartilage [157].

### Limitations of Using Nanoparticles in Tissue Engineering

In recent years, advances in nanotechnology have led to significant changes in tissue engineering, including the development of smart drugs, the repair and reconstruction of injured tissues [158,159]. In nanotechnology, tissues can be engineered at the nanoscale, allowing complex three-dimensional architectures to be developed. It is also possible to precisely control material properties, such as stiffness, elasticity, and permeability, which are important for tissue engineering [111,160]. It is therefore essential to choose the appropriate nanomaterial for different applications due to tissue heterogeneity [161,162]. There are still many challenges to overcome in order to make them available for clinical use in large numbers. The limitations of nanotechnology in tissue engineering are the lack of understanding of complex interactions between biomaterials and cells, the difficulty of controlling the precise arrangement of biomaterials and cells, and the difficulty in reproducing nanoscale features in tissues [163,164,165]. These limitations are due to the fact that the nanoscale features of tissues are very complex, and the interactions between cells and biomaterials are not well understood [149,161]. While using these nanoparticles for certain period it might lead to accumulation in body which could cause negative impact [166,167,168]. Still more research on cytotoxicity, nanoparticle accumulation, and early-stage regulatory criteria are required [169]. The precautionary principle should be followed during the development, testing, and clinical application of these materials. There is still a long way to go in the biosafety, utilization, and stability of nanomaterials [163,170,171]. 

## 6. Advantages and Disadvantages of Using Biopolymers in Scaffold Synthesis

The use of biopolymers, with the parameters of wall morphology, pore size and shape, pore interconnectivity, porosity, and surface area, allows scaffolds to be manufactured with the characteristics of better cell seeding, migration, growth, mass transport, and tissue development [172]. In addition, the usage of natural polymers in scaffold synthesis includes biological renewability, biodegradability, biocompatibility, nonantigenicity, nontoxicity, biofunctionality, acting as good bioadhesive material, improved cellular interaction, and good cell recognition [173,174]. In contrast, the disadvantages of using natural polymers include poor mechanical properties, rapid degradation in-vivo, difficulty in maintaining structural integrity, etc. [174,175].

## 7. Conclusions

Generally, natural biodegradable polymeric materials have the essential physicochemical, biological, and mechanical capabilities to make them suitable candidates for tissue engineering. Additionally, they possess a number of special qualities, like biocompatibility, absorbability, and bioavailability. They can be used as scaffolds, sutures for the regeneration of new tissue, tissue adhesives, hemostats, and other tissue engineering-related devices, making them a superior alternative to traditional scaffolds. In the future, the main focus will be drug delivery scaffolds, which are considered to be innovative substitutes for traditional formulations that enable regulated spatiotemporal releases of active ingredients, which includes the usage of both natural and synthetic polymers.

## Data Availability

The data used to support the findings of this study are included in the article. Should further data or information be required, these are available from the corresponding author upon request.

## References

[1] Zhao Y., Song S., Ren X., Zhang J., Lin Q., Zhao Y. (2022). Supramolecular Adhesive Hydrogels for Tissue Engineering Applications. Chem. Rev..

[2] Mhanna R., Hasan A., Hasan A. (2017). Introduction to tissue engineering. Tissue Engineering for Artificial Organs: Regenerative Medicine; Smart Diagnostics and Personalized Medicine.

[3] Pancrazio J.J., Wang F., Kelley C.A. (2007). Enabling tools for tissue engineering. Biosens. Bioelectron..

[4] Huang L., Abdalla A.M.E., Xiao L., Yang G. (2020). Biopolymer-Based Microcarriers for Three-Dimensional Cell Culture and Engineered Tissue Formation. Int. J. Mol. Sci..

[5] Do A.V., Khorsand B., Geary S.M., Salem A.K. (2015). 3D printing of scaffolds for tissue regeneration applications. Adv. Healthc. Mater..

[6] Ahmad A., Younas M., Santulli C., Kiyani M.Z., Ali R., Habbiba O., Zubair M. (2021). Nanomedicine and tissue engineering. Nanomed. Manuf. Appl..

[7] Kass L.E., Nguyen J. (2022). Nanocarrier-hydrogel composite delivery systems for precision drug release. Interdiscip. Rev. Nanomed. Nanobiotechnol..

[8] Armentano I., Dottori M., Fortunati E., Mattioli S., Kenny J.M. (2010). Biodegradable polymer matrix nanocomposites for tissue engineering: A review. Polym. Degrad. Stab..

[9] Zulkiflee I., Fauzi M.B. (2021). Gelatin-polyvinyl alcohol film for tissue engineering: A concise review. Biomedicines.

[10] Putra R.U., Basri H., Prakoso A.T., Chandra H., Ammarullah M.I., Akbar I., Syahrom A., Kamarul T. (2023). Level of Activity Changes Increases the Fatigue Life of the Porous Magnesium Scaffold, as Observed in Dynamic Immersion Tests, over Time. Sustainability.

[11] Polo-Corrales L., Latorre-Esteves M., Ramirez-Vick J.E. (2014). Scaffold design for bone regeneration. J. Nanosci. Nanotechnol..

[12] Fard M.G., Sharifianjazi F., Kazemi S.S., Rostamani H., Bathaei M.S. (2022). Laser-Based Additive Manufacturing of Magnesium Alloys for Bone Tissue Engineering Applications: From Chemistry to Clinic. J. Manuf. Mater. Process..

[13] Du J., Hu X., Su Y., Wei T., Jiao Z., Liu T., Wang H., Nie Y., Li X., Song K. (2022). Gelatin/sodium alginate hydrogel-coated decellularized porcine coronary artery to construct bilayer tissue engineered blood vessels. Int. J. Biol. Macromol..

[14] Ikada Y. (2006). Challenges in tissue engineering. J. R. Soc. Interface.

[15] Chan B.P., Leong K. (2008). Scaffolding in tissue engineering: General approaches and tissue-specific considerations. Eur. Spine J. Off. Publ. Eur. Spine Soc. Eur. Spinal Deform. Soc. Eur. Sect. Cerv. Spine Res. Soc..

[16] Chen J., Zhang X., Cai H., Chen Z., Wang T., Jia L., Wang J., Wan Q., Pei X. (2016). Osteogenic activity and antibacterial effect of zinc oxide/carboxylated graphene oxide nanocomposites: Preparation and in vitro evaluation. Colloids Surf. B Biointerfaces.

[17] Ammarullah M.I., Santoso G., Sugiharto S., Supriyono T., Wibowo D.B., Kurdi O., Tauviqirrahman M., Jamari J. (2022). Minimizing Risk of Failure from Ceramic-on-Ceramic Total Hip Prosthesis by Selecting Ceramic Materials Based on Tresca Stress. Sustainability.

[18] Fergal J. (2011). O’Brien, Biomaterials & scaffolds for tissue engineering. Mater. Today.

[19] Dhandayuthapani B., Yasuhiko Y., Maekawa T., Sakthi K.D. (2011). Polymeric Scaffolds in Tissue Engineering Application: A Review. Int. J. Polym. Sci..

[20] Jones J.R. (2005). Scaffolds for tissue engineering. Biomaterials; Artificial Organs and Tissue Engineering; Woodhead Publishing Series in Biomaterials.

[21] Song R., Murphy M., Li C., Ting K., Soo C., Zheng Z. (2018). Current development of biodegradable polymeric materials for biomedical applications. Drug Des. Dev. Ther..

[22] Bordbar-Khiabani A., Gasik M. (2022). Smart Hydrogels for Advanced Drug Delivery Systems. Int. J. Mol. Sci..

[23] Ulery B.D., Nair L.S., Laurencin C.T. (2011). Biomedical Applications of Biodegradable Polymers. J. Polym. Science. Part B Polym. Phys..

[24] Hutmacher D.W., Goh J.C.H., Teoh S.H. (2001). An introduction to biodegradable materials for tissue engineering applications. Ann. Acad. Med. Singap..

[25] Ren X., Zhao M., Lash B., Martino M.M., Julier Z. (2020). Growth factor engineering strategies for regenerative medicine applications. Front. Bioeng. Biotechnol..

[26] Berthiaume F., Yarmush M.L. (2003). Tissue Engineering. Encyclopedia of Physical Science and Technology.

[27] Bauer S., Schmuki P., Von Der Mark K., Park J. (2013). Engineering biocompatible implant surfaces: Part I: Materials and surfaces. Prog. Mater. Sci..

[28] Samrot A.V., Samanvitha S.K., Shobana N., Renitta E.R., Senthilkumar P., Kumar S.S., Abirami S., Dhiva S., Bavanilatha M., Prakash P. (2021). The synthesis, characterization and applications of polyhydroxyalkanoates (PHAs) and PHA-based nanoparticles. Polymers.

[29] Pachiyappan S., Shanmuganatham S.D., Kuppa S.S., Chandrasekaran S., Samrot A.V. (2019). Surfactant-mediated synthesis of polyhydroxybutyrate (PHB) nanoparticles for sustained drug delivery. IET Nanobiotechnol..

[30] Samrot A.V., Shobana N., Philip S.A., Burman U., Chandrasekaran K. (2018). Utilization of crab shell derived chitosan for production of gallic acid loaded nanocomposites for drug delivery. J. Pharm. Sci. Res..

[31] Shobana N., Kumar P.S., Raji P., Samrot A.V. (2019). Utilization of crab shell-derived chitosan in nanoparticle synthesis for Curcumin delivery. Indian J. Geo Mar. Sci..

[32] Samrot A.V., Burman U., Philip S.A., Shobana N., Chandrasekaran K. (2018). Synthesis of curcumin loaded polymeric nanoparticles from crab shell derived chitosan for drug delivery. Inform. Med. Unlocked.

[33] Bulte J.W., Douglas T., Witwer B., Zhang S.C., Strable E., Lewis B.K., Zywicke H., Miller B., Van Gelderen P., Moskowitz B.M. (2001). Magnetodendrimers allow endosomal magnetic labeling and in-vivo tracking of stem cells. Nat. Biotechnol..

[34] Murugan R., Ramakrishna S. (2005). Development of nanocomposites for bone grafting. Compos. Sci. Technol..

[35] Hule R.A., Pochan D.J. (2007). Polymer nanocomposites for biomedical applications. MRS Bull..

[36] Tiwari S., Patil R., Bahadur P. (2018). Polysaccharide Based Scaffolds for Soft Tissue Engineering Applications. Polymers.

[37] Jin M., Shi J., Zhu W., Yao H., Wang D. (2020). Polysaccharide-based biomaterials in tissue engineering: A review. Tissue Eng. Part B Rev..

[38] Naumenko E.A., Guryanov I.D., Yendluri R., Lvov Y.M., Fakhrullin R.F. (2016). Clay nanotube–biopolymer composite scaffolds for tissue engineering. Nanoscale.

[39] Sheeny K., Zhang L.M.L. (2014). Chitosan-based scaffolds for bone tissue engineering. J. Mater. Chem. B.

[40] Von Palubitzki L., Wang Y., Hoffmann S., Vidal-y-Sy S., Zobiak B., Failla A.V., Schmage P., John A., Osorio-Madrazo A., Bauer A.T. (2020). Differences of the tumour cell glycocalyx affect binding of capsaicin-loaded chitosan nanocapsules. Sci. Rep..

[41] Yang B., Li X., Shi S., Kong X., Guo G., Huang M., Luo F., Wei Y., Zhao X., Qian Z. (2010). Preparation and characterization of a novel chitosan scaffold. Carbohydr. Polym..

[42] Saravanan S., Leena R.S., Selvamurugan N. (2016). Chitosan based biocomposite scaffolds for bone tissue engineering. Int. J. Biol. Macromol..

[43] Sasaki T., Iwasaki N., Kohno K., Kishimoto M., Majima T., Nishimura S.I., Minami A. (2008). Magnetic nanoparticles for improving cell invasion in tissue engineering. J. Biomed. Mater. Res. Part A.

[44] Abd-Khorsand S., Saber-Samandari S., Saber-Samandari S. (2017). Development of nanocomposite scaffolds based on TiO_2_ doped in grafted chitosan/hydroxyapatite by freeze drying method and evaluation of biocompatibility. Int. J. Biol. Macromol..

[45] Aktug S.L., Durdu S., Kalkan S., Cavusoglu K., Usta M. (2021). In Vitro biological and antimicrobial properties of chitosan-based bioceramic coatings on zirconium. Sci. Rep..

[46] Naseri N., Poirier J.M., Girandon L., Frohlich M., Oksman K., Mathew A.P. (2016). 3-Dimensional porous nanocomposite scaffolds based on cellulose nanofibers for cartilage tissue engineering: Tailoring of porosity and mechanical performance. RSC Adv..

[47] Atiqah A., Ansari M.N.M. (2019). Nanostructure–Polymer Composites for Soft-Tissue Engineering. Nanostruct. Polym. Compos. Biomed. Appl..

[48] Parajó Y., d’Angelo I., Welle A., Garcia-Fuentes M., Alonso M.J. (2010). Hyaluronic acid/Chitosan nanoparticles as delivery vehicles for VEGF and PDGF-BB. Drug Deliv..

[49] Muzzarelli R.A.A., Mattioli-Belmonte M., Tietz C., Biagini R., Ferioli G., Brunelli M.A., Biagini G. (1994). Stimulatory effect on bone formation exerted by a modified chitosan. Biomaterials.

[50] Zhu Y., Zhang Y., Zhou Y. (2022). Application Progress of Modified Chitosan and Its Composite Biomaterials for Bone Tissue Engineering. Int. J. Mol. Sci..

[51] Pezeshki-Modaress M., Rajabi-Zeleti S., Zandi M., Mirzadeh H., Sodeifi N., Nekookar A., Aghdami N. (2013). Cell-loaded gelatin/chitosan scaffolds fabricated by salt-leaching/lyophilization for skin tissue engineering: In Vitro and in vivo study. J. Biomed. Mater. Res. Part A.

[52] Refifi J., Oudadesse H., Merdrignac-Conanec O., El Feki H., Lefeuvre B. (2020). Salt leaching using powder (SLUP) process for glass/chitosan scaffold elaboration for biomaterial applications. J. Aust. Ceram. Soc..

[53] Sukpaita T., Chirachanchai S., Pimkhaokham A., Ampornaramveth R.S. (2021). Chitosan-Based Scaffold for Mineralized Tissues Regeneration. Mar. Drugs.

[54] Seddighian A., Ganji F., Baghaban-Eslaminejad M., Bagheri F. (2021). Electrospun PCL scaffold modified with chitosan nanoparticles for enhanced bone regeneration. Prog. Biomater..

[55] Mad Jin R., Sultana N., Baba S., Hamdan S., Ismail A.F. (2015). Porous PCL/chitosan and nHA/PCL/chitosan scaffolds for tissue engineering applications: Fabrication and evaluation. J. Nanomater..

[56] Amini A.R., Laurencin C.T., Nukavarapu S.P. (2012). Bone tissue engineering: Recent advances and challenges. Crit. Rev. Biomed. Eng..

[57] Rodríguez-Vázquez M., Vega-Ruiz B., Ramos-Zúñiga R., Saldaña-Koppel D.A., Quiñones-Olvera L.F. (2015). Chitosan and its potential use as a scaffold for tissue engineering in regenerative medicine. BioMed Res. Int..

[58] Yamada Y., Yoshida C., Hamada K., Kikkawa Y., Nomizu M. (2020). Development of Three-Dimensional Cell Culture Scaffolds using Laminin Peptide-Conjugated Agarose Microgels. Biomacromolecules.

[59] Irastorza-Lorenzo A., Sánchez-Porras D., Ortiz-Arrabal O., de Frutos M.J., Esteban E., Fernández J., Janer A., Campos A., Campos F., Alaminos M. (2021). Evaluation of Marine Agarose Biomaterials for Tissue Engineering Applications. Int. J. Mol. Sci..

[60] Ali A.F., Ahmed M.M., El-Kady A.M., Abd El-Hady B.M., Ibrahim A.M. (2020). Synthesis of Gelatin-Agarose Scaffold for Controlled Antibiotic Delivery and its Modification by Glass Nanoparticles Addition as a Potential Osteomyelitis Treatment. Silicon.

[61] Salati M.A., Khazai J., Tahmuri A.M., Samadi A., Taghizadeh A., Taghizadeh M., Zarrintaj P., Ramsey J.D., Habibzadeh S., Seidi F. (2020). Agarose-based biomaterials: Opportunities and challenges in cartilage tissue engineering. Polymers.

[62] Varoni E., Tschon M., Palazzo B., Nitti P., Martini L., Rimondini L. (2012). Agarose Gel as Biomaterial or Scaffold for Implantation Surgery: Characterization; Histological and Histomorphometric Study on Soft Tissue Response. Connect. Tissue Res..

[63] Cigan A.D., Roach B.L., Nims R.J., Tan A.R., Albro M.B., Stoker A.M., Cook J.L., Vunjak-Novakovic G., Hung C.T., Ateshian G.A. (2016). High seeding density of human chondrocytes in agarose produces tissue-engineered cartilage approaching native mechanical and biochemical properties. J. Biomech..

[64] Xu G., Yin F., Wu H., Hu X., Zheng L., Zhao J. (2014). In Vitro ovarian cancer model based on three-dimensional agarose hydrogel. J. Tissue Eng..

[65] Campos F., Bonhome-Espinosa A.B., Chato-Astrain J., Sánchez-Porras D., García-García Ó.D., Carmona R., López-López M.T., Alaminos M., Carriel V., Rodriguez I.A. (2020). Evaluation of fibrin-agarose tissue-like hydrogels biocompatibility for tissue engineering applications. Front. Bioeng. Biotechnol..

[66] Singh Y.P., Bhardwaj N., Mandal B.B. (2016). Potential of agarose/silk fibroin blended hydrogel for in vitro cartilage tissue engineering. ACS Appl. Mater. Interfaces.

[67] Mauck R.L., Soltz M.A., Wang C.C., Wong D.D., Chao P.-H.G., Valhmu W.B., Hung C.T., Ateshian G.A. (2000). Functional tissue engineering of articular cartilage through dynamic loading of chondrocyte-seeded agarose gels. J. Biomech. Eng..

[68] Khosalim I.P., Zhang Y.Y., Yiu C.K.Y., Wong H.M. (2022). Synthesis of a graphene oxide/agarose/hydroxyapatite biomaterial with the evaluation of antibacterial activity and initial cell attachment. Sci. Rep..

[69] Wang B., Guo Y., Chen X., Zeng C., Hu Q., Yin W., Li W., Xie H., Zhang B., Huang X. (2018). Nanoparticle-modified chitosan-agarose-gelatin scaffold for sustained release of SDF-1 and BMP-2. Int. J. Nanomed..

[70] Utech S., Boccaccini A.R. (2016). A review of hydrogel-based composites for biomedical applications: Enhancement of hydrogel properties by addition of rigid inorganic fillers. J. Mater. Sci..

[71] Liu W., Du H., Zhang M., Liu K., Liu H., Xie H., Zhang X., Si C. (2020). Bacterial cellulose-based composite scaffolds for biomedical applications: A review. ACS Sustain. Chem. Eng..

[72] Joseph B., Sagarika V.K., Sabu C., Kalarikkal N., Thomas S. (2020). Cellulose nanocomposites: Fabrication and biomedical applications. J. Bioresour. Bioprod..

[73] Tamo A.K., Tran T.A., Doench I., Jahangir S., Lall A., David L., Peniche-Covas C., Walther A., Osorio-Madrazo A. (2022). 3D Printing of Cellulase-Laden Cellulose Nanofiber/Chitosan Hydrogel Composites: Towards Tissue Engineering Functional Biomaterials with Enzyme-Mediated Biodegradation. Materials.

[74] Kamdem Tamo A., Doench I., Walter L., Montembault A., Sudre G., David L., Morales-Helguera A., Selig M., Rolauffs B., Bernstein A. (2021). Development of Bioinspired Functional Chitosan/Cellulose Nanofiber 3D Hydrogel Constructs by 3D Printing for Application in the Engineering of Mechanically Demanding Tissues. Polymers.

[75] Ali S.W., Chowdhury A., Nath J., Dolui S.K., Gadkari R.R. (2021). Cellulose-based bionanocomposites in tissue engineering and regenerative medicine. Bionanocomposites in Tissue Engineering and Regenerative Medicine.

[76] Abdelhamid H.N., Mathew A.P. (2022). Cellulose-Based Nanomaterials Advance Biomedicine: A Review. Int. J. Mol. Sci..

[77] Hickey R.J., Pelling A.E. (2019). Cellulose Biomaterials for Tissue Engineering. Front. Bioeng. Biotechnol..

[78] Janmohammadi M., Nazemi Z., Salehi A.O.M., Seyfoori A., John J.V., Nourbakhsh M.S., Akbari M. (2022). Cellulose-based composite scaffolds for bone tissue engineering and localized drug delivery. Bioact Mater..

[79] Ostrakhovitch E.A., Byers J.C., O’Neil K.D., Semenikhin O.A. (2012). Directed differentiation of embryonic P19 cells and neural stem cells into neural lineage on conducting PEDOT-PEG and ITO glass substrates. Arch Biochem. Biophys..

[80] Kuzmenko V., Kalogeropoulos T., Thunberg J., Johannesson S., Hägg D., Enoksson P., Gatenholm P. (2016). Enhanced growth of neural networks on conductive cellulose-derived nanofibrous scaffolds. Mater. Sci. Eng. C.

[81] Novotna K., Havelka P., Sopuch T., Kolarova K., Vosmanska V., Lisa V., Svorcik V., Bacakova L. (2013). Cellulose-based materials as scaffolds for tissue engineering. Cellulose.

[82] Alihosseini F. (2016). Plant-based compounds for antimicrobial textiles. Antimicrobial Textiles.

[83] Szekalska M., Puciłowska A., Szymańska E., Ciosek P., Winnicka K. (2016). Alginate: Current use and future perspectives in pharmaceutical and biomedical applications. Int. J. Polym. Sci..

[84] Lee K.Y., Mooney D.J. (2012). Alginate: Properties and biomedical applications. Prog. Polym. Sci..

[85] Abasalizadeh F., Moghaddam S.V., Alizadeh E., Akbari E., Kashani E., Fazljou S.M.B., Torbati M., Akbarzadeh A. (2020). Alginate-based hydrogels as drug delivery vehicles in cancer treatment and their applications in wound dressing and 3D bioprinting. J. Biol. Eng..

[86] Mollah M.Z.I., Zahid H.M., Mahal Z., Faruque M.R.I., Khandaker M.U. (2021). The Usages and Potential Uses of Alginate for Healthcare Applications. Front. Mol. Biosci..

[87] Farokhi M., Jonidi Shariatzadeh F., Solouk A., Mirzadeh H. (2019). Alginate Based Scaffolds for Cartilage Tissue Engineering: A Review. Int. J. Polym. Mater. Polym. Biomater..

[88] Naghieh S., Karamooz-Ravari M.R., Sarker M.D., Karki E., Chen X. (2018). Influence of Crosslinking on the Mechanical Behavior of 3D Printed Alginate Scaffolds: Experimental and Numerical Approaches. J. Mech. Behav. Biomed. Mater..

[89] Sahoo D.R., Biswal T. (2021). Alginate and its application to tissue engineering. SN Appl. Sci..

[90] Zahedi P., Rezaeian I., Ranaei-Siadat S.-O., Jafari S.-H., Supaphol P. (2009). A review on wound dressings with an emphasis on electrospun nanofibrous polymeric bandages. Polym. Adv. Technol..

[91] Sun J., Tan H. (2013). Alginate-Based Biomaterials for Regenerative Medicine Applications. Materials.

[92] Díaz-Montes E. (2021). Dextran: Sources; structures; and properties. Polysaccharides.

[93] Sarwat F., Qader S.A.U., Aman A., Ahmed N. (2008). Production & characterization of a unique dextran from an indigenous *Leuconostoc mesenteroides* CMG713. Int. J. Biol..

[94] Sun G., Mao J.J. (2012). Engineering dextran-based scaffolds for drug delivery and tissue repair. Nanomedicine.

[95] Banerjee S., Szepes M., Dibbert N., Rios-Camacho J.C., Kirschning A., Gruh I., Dräger G. (2021). Dextran-based scaffolds for in-situ hydrogelation: Use for next generation of bioartificial cardiac tissues. Carbohydr. Polym..

[96] Lévesque S.G., Lim R.M., Shoichet M.S. (2005). Macroporous interconnected dextran scaffolds of controlled porosity for tissue-engineering applications. Biomaterials.

[97] Maia J., Evangelista M.B., Gil H., Ferreira L. (2014). Dextran-based materials for biomedical applications. Res. Signpost.

[98] Varghese S.A., Rangappa S.M., Siengchin S., Parameswaranpillai J. (2020). Natural polymers and the hydrogels prepared from them. Hydrogels Based on Natural Polymers.

[99] Jahangirian H., Azizi S., Rafiee-Moghaddam R., Baratvand B., Webster T.J. (2019). Status of Plant Protein-Based Green Scaffolds for Regenerative Medicine Applications. Biomolecules.

[100] Iravani S., Varma R.S. (2019). Plants and plant-based polymers as scaffolds for tissue engineering. Green Chem..

[101] Wang H.J., Di L., Ren Q.S., Wang J.Y. (2009). Applications and Degradation of Proteins Used as Tissue Engineering Materials. Materials.

[102] Chien K.B., Shah R.N. (2012). Novel soy protein scaffolds for tissue regeneration: Material characterization and interaction with human mesenchymal stem cells. Acta Biomater..

[103] Nitta S., Numata K. (2013). Biopolymer-Based Nanoparticles for Drug/Gene Delivery and Tissue Engineering. Int. J. Mol. Sci..

[104] Dong C., Lv Y. (2016). Application of Collagen Scaffold in Tissue Engineering: Recent Advances and New Perspectives. Polymers.

[105] Glowacki J., Mizuno S. (2008). Collagen scaffolds for tissue engineering. Biopolymers.

[106] Mizuno S., Glowacki J. (1996). Three-dimensional composite of demineralized bone powder and collagen for in vitro analysis of chondroinduction of human dermal fibroblasts. Biomaterials.

[107] Percival N.J. (2002). Classification of wounds and their management. Surgery.

[108] Palmero P. (2016). Ceramic–polymer nanocomposites for bone-tissue regeneration. Nanocompos. Musculoskelet. Tissue Regen..

[109] Itoh S., Kikuchi M., Takakuda K., Koyama Y., Matsumoto H.N., Ichinose S., Tanaka J., Kawauchi T., Shinomiya K. (2001). The biocompatibility and osteoconductive activity of a novel hydroxyapatite/collagen composite biomaterial, and its function as a carrier of rhBMP-2. J. Biomed. Mater. Res. Off. J. Soc. Biomater. Jpn. Soc. Biomater..

[110] Gholami A., Hashemi S.A., Yousefi K., Mousavi S.M., Chiang W.H., Ramakrishna S., Mazraedoost S., Alizadeh A., Omidifar N., Behbudi G. (2020). 3D Nanostructures for Tissue Engineering; Cancer Therapy; and Gene Delivery. J. Nanomater..

[111] Fathi-Achachelouei M., Knopf-Marques H., Ribeiro da Silva C.E., Barthès J., Bat E., Tezcaner A., Vrana N.E. (2019). Use of Nanoparticles in Tissue Engineering and Regenerative Medicine. Front. Bioeng. Biotechnol..

[112] Silva S., Oliveira J.M., Mano J.F., Reis R.L. (2006). Physicochemical characterization of novel chitosan-soy protein/TEOS porous hybrids for tissue engineering applications. Mater. Sci. Forum.

[113] Kokubo T. (1998). Apatite formation on surfaces of ceramics; metals and polymers in body environment. Acta Mater..

[114] Zhao Y., He M., Zhao L., Wang S., Li Y., Gan L., Li M., Xu L., Chang P.R., Anderson D.P. (2016). Epichlorohydrin-cross-linked hydroxyethyl cellulose/soy protein isolate composite films as biocompatible and biodegradable implants for tissue engineering. ACS Appl. Mater. Interfaces.

[115] Zhang X., Dong C., Hu Y., Gao M., Luan G. (2021). Zein as a structural protein in gluten-free systems: An overview. Food Sci. Hum. Wellness.

[116] Plath A.M.S., Facchi S.P., Souza P.R., Sabino R.M., Corradini E., Muniz E.C., Popat K.C., Kipper M.J., Martins A.F. (2021). Zein supports scaffolding capacity toward mammalian cells and bactericidal and antiadhesive properties on poly(ε-caprolactone)/zein electrospun fibers. Mater. Today Chem..

[117] Pedram Rad Z., Mokhtari J., Abbasi M. (2018). Fabrication and characterization of PCL/zein/gum arabic electrospun nanocomposite scaffold for skin tissue engineering. Mater. Sci. Eng. C Mater. Biol. Appl..

[118] Pérez-Guzmán C.J., Castro-Muñoz R. (2020). A Review of Zein as a Potential Biopolymer for Tissue Engineering and Nanotechnological Applications. Processes.

[119] Liu C., Yang H., Shen N.A., Li J., Chen Y., Wang J.Y. (2021). Improvement of mechanical properties of zein porous scaffold by quenching/electrospun fiber reinforcement. Biomed. Mater..

[120] Tortorella S., Maturi M., Buratti V.V., Vozzolo G., Locatelli E., Sambri L., Franchini M.C. (2021). Zein as a versatile biopolymer: Different shapes for different biomedical applications. RSC Adv..

[121] Ghosh S., Webster T.J. (2021). Metallic Nanoscaffolds as Osteogenic Promoters: Advances; Challenges and Scope. Metals.

[122] Corradini E., Curti P.S., Meniqueti A.B., Martins A.F., Rubira A.F., Muniz E.C. (2014). Recent advances in food-packing, pharmaceutical and biomedical applications of zein and zein-based materials. Int. J. Mol. Sci..

[123] Zein N., Harmouch E., Lutz J.C., Fernandez De Grado G., Kuchler-Bopp S., Clauss F., Offner D., Hua G., Benkirane-Jessel N., Fioretti F. (2019). Polymer-based instructive scaffolds for endodontic regeneration. Materials.

[124] Putri T.S., Ratnasari A., Sofiyaningsih N., Nizar M.S., Yuliati A., Shariff K.A. (2022). Mechanical improvement of chitosan–gelatin scaffolds reinforced by β-tricalcium phosphate bioceramic. Ceram. Int..

[125] Eivazzadeh-Keihan R., Bahojb Noruzi E., Khanmohammadi Chenab K., Jafari A., Radinekiyan F., Hashemi S.M., Ahmadpour F., Behboudi A., Mosafer J., Mokhtarzadeh A. (2020). Metal-based nanoparticles for bone tissue engineering. J. Tissue Eng. Regen. Med..

[126] Dhivya S., Ajita J., Selvamurugan N. (2015). Metallic Nanomaterials for Bone Tissue Engineering. J. Biomed. Nanotechnol..

[127] Bianchi E., Vigani B., Viseras C., Ferrari F., Rossi S., Sandri G. (2022). Inorganic Nanomaterials in Tissue Engineering. Pharmaceutics.

[128] Samrot A.V., Sai Bhavya K., Sruthi P.D., Paulraj P. (2020). Synthesis of SPIONs to deliver drug in-vitro and to use as contrasting agent. Int. J. Adv. Res. Eng. Technol..

[129] Samrot A.V., Saipriya C., Durga S.P., Selvarani A.J., Raji P., Prakash P., Ponnaiah P., Thirumurugan R., Pattammadath S., Purayil S.K. (2020). Production and Utilization of SPIONs for in-vitro Drug Release and X-ray Imaging. J. Pure Appl. Microbiol..

[130] Justin C., Philip S.A., Samrot A.V. (2017). Synthesis and characterization of superparamagnetic iron-oxide nanoparticles (SPIONs) and utilization of SPIONs in X-ray imaging. Appl. Nanosci..

[131] Justin C., Samrot A.V., Sahithya C.S., Bhavya K.S., Saipriya C. (2018). Preparation, characterization and utilization of coreshell super paramagnetic iron oxide nanoparticles for curcumin delivery. PLoS ONE.

[132] Mok H., Zhang M. (2013). Superparamagnetic iron oxide nanoparticle-based delivery systems for biotherapeutics. Expert Opin. Drug Deliv..

[133] Fan D., Wang Q., Zhu T., Wang H., Liu B., Wang Y., Liu Z., Liu X., Fan D., Wang X. (2020). Recent advances of magnetic nanomaterials in bone tissue repair. Front. Chem..

[134] Ito A., Honda H., Kamihira M. (2008). Construction of 3D Tissue-Like Structure Using Functional Magnetite Nanoparticles. Yakugaku Zasshi.

[135] Kaczyńska A., Guzdek K., Derszniak K., Karewicz A., Lewandowska-Łańcucka J., Mateuszuk Ł., Skórka T., Banasik T., Jasiński K., Kapusta C. (2016). Novel nanostructural contrast for magnetic resonance imaging of endothelial inflammation: Targeting SPIONs to vascular endothelium. RSC Adv..

[136] Gao X.H., Cui Y.Y., Levenson R.M., Chung L.W.K., Nie S.M. (2004). In-Vivo cancer targeting and imaging with semiconductor quantum dots. Nat. Biotechnol..

[137] Yudintceva N., Lomert E., Mikhailova N., Tolkunova E., Agadzhanian N., Samochernych K., Multhoff G., Timin G., Ryzhov V., Deriglazov V. (2021). Targeting Brain Tumors with Mesenchymal Stem Cells in the Experimental Model of the Orthotopic Glioblastoma in Rats. Biomedicines.

[138] Dang M., Saunders L., Niu X., Fan Y., Ma P.X. (2018). Biomimetic delivery of signals for bone tissue engineering. Bone Res..

[139] Bramhill J., Ross S., Ross G. (2017). Bioactive Nanocomposites for Tissue Repair and Regeneration: A Review. Int. J. Environ. Res. Public Health.

[140] Sanz-Herrera J.A. (2020). Biomaterials for Bone Tissue Engineering.

[141] Li D., Liu T., Yu X., Wu D., Su Z. (2017). Fabrication of graphene–biomacromolecule hybrid materials for tissue engineering application. Polym. Chem..

[142] Edwards S.L., Werkmeister J.A., Ramshaw J.A. (2009). Carbon nanotubes in scaffolds for tissue engineering. Expert Rev. Med. Devices.

[143] Bahrami S., Baheiraei N., Shahrezaee M. (2021). Biomimetic reduced graphene oxide coated collagen scaffold for in situ bone regeneration. Sci. Rep..

[144] Parvez K. (2019). Two-Dimensional Nanomaterials: Crystal Structure and Synthesis. Biomed. Appl. Graphene 2d Nanomater..

[145] Chimene D., Alge D.L., Gaharwar A.K. (2015). Two-Dimensional Nanomaterials for Biomedical Applications: Emerging Trends and Future Prospects. Adv. Mater..

[146] Wu X., Jiang X., Fan T., Zheng Z., Liu Z., Chen Y., Cao L., Xie Z., Zhang D., Zhao J. (2020). Recent advances in photodynamic therapy based on emerging two-dimensional layered nanomaterials. Nano Res..

[147] Derakhshi M., Daemi S., Shahini P., Habibzadeh A., Mostafavi E., Ashkarran A.A. (2022). Two-Dimensional Nanomaterials beyond Graphene for Biomedical Applications. J. Funct. Biomater..

[148] Jing X., Mi H.Y., Napiwocki B.N., Peng X.F., Turng L.S. (2017). Mussel-inspired electroactive chitosan/graphene oxide composite hydrogel with rapid self-healing and recovery behavior for tissue engineering. Carbon.

[149] Zheng Y., Hong X., Wang J., Feng L., Fan T., Guo R., Zhang H. (2021). 2D Nanomaterials for Tissue Engineering and Regenerative Nanomedicines: Recent Advances and Future Challenges. Adv. Healthc. Mater..

[150] Magaz A., Li X., Gough J.E., Blaker J.J. (2021). Graphene oxide and electroactive reduced graphene oxide-based composite fibrous scaffolds for engineering excitable nerve tissue. Mater. Sci. Eng. C.

[151] Nayak T.R., Andersen H., Makam V.S., Khaw C., Bae S., Xu X., Ee P.L.R., Ahn J.H., Hong B.H., Pastorin G. (2011). Graphene for controlled and accelerated osteogenic differentiation of human mesenchymal stem cells. ACS Nano.

[152] Geetha Bai R., Muthoosamy K., Manickam S., Hilal-Alnaqbi A. (2019). Graphene-based 3D scaffolds in tissue engineering: Fabrication, applications, and future scope in liver tissue engineering. Int. J. Nanomed..

[153] Lee J.H., Shin Y.C., Lee S.M., Jin O.S., Kang S.H., Hong S.W., Jeong C.M., Huh J.B., Han D.W. (2015). Enhanced osteogenesis by reduced graphene oxide/hydroxyapatite nanocomposites. Sci. Rep..

[154] Jabbari F., Hesaraki S., Houshmand B. (2019). The physical, mechanical and biological properties of silk fibroin/chitosan/reduced graphene oxide composite membranes for guided bone regeneration. J. Biomat. Sci. Polym. Ed..

[155] Zhang J., Chen H., Zhao M., Liu G., Wu J. (2020). 2D nanomaterials for tissue engineering application. Nano Res..

[156] Rafiei-Sarmazdeh Z., Morteza Zahedi-Dizaji S., Kafi Kang A. (2020). Two-Dimensional Nanomaterials. Nanostructures.

[157] Zhao Y., Wei C., Chen X., Liu J., Yu Q., Liu Y., Liu J. (2019). Drug Delivery System Based on Near-Infrared Light-Responsive Molybdenum Disulfide Nanosheets Controls the High-Efficiency Release of Dexamethasone to Inhibit Inflammation and Treat Osteoarthritis. ACS Appl. Mater. Interfaces.

[158] Shi J., Votruba A.R., Farokhzad O.C., Langer R. (2010). Nanotechnology in drug delivery and tissue engineering: From discovery to applications. Nano Lett..

[159] Saghazadeh S., Rinoldi C., Schot M., Kashaf S.S., Sharifi F., Jalilian E., Nuutila K., Giatsidis G., Mostafalu P., Derakhshandeh H. (2018). Drug delivery systems and materials for wound healing applications. Adv. Drug Deliv. Rev..

[160] Saydé T., El Hamoui O., Alies B., Gaudin K., Lespes G., Battu S. (2021). Biomaterials for Three-Dimensional Cell Culture: From Applications in Oncology to Nanotechnology. Nanomaterials.

[161] Zheng X., Zhang P., Fu Z., Meng S., Dai L., Yang H. (2021). Applications of nanomaterials in tissue engineering. RSC Adv..

[162] Mitchell M.J., Billingsley M.M., Haley R.M., Wechsler M.E., Peppas N.A., Langer R. (2021). Engineering precision nanoparticles for drug delivery. Nat. Rev. Drug Discov..

[163] Hasan A., Morshed M., Memic A., Hassan S., Webster T.J., Marei H.E. (2018). Nanoparticles in tissue engineering: Applications, challenges and prospects. Int. J. Nanomed..

[164] Desai N. (2012). Challenges in development of nanoparticle-based therapeutics. AAPS J..

[165] Troy E., Tilbury M.A., Power A.M., Wall J.G. (2021). Nature-based biomaterials and their application in biomedicine. Polymers.

[166] Gupta R., Xie H. (2018). Nanoparticles in Daily Life: Applications, Toxicity and Regulations. J. Environ. Pathol. Toxicol. Oncol. Off. Organ Int. Soc. Environ. Toxicol. Cancer.

[167] Chenthamara D., Subramaniam S., Ramakrishnan S.G., Krishnaswamy S., Essa M.M., Lin F.H., Qoronfleh M.W. (2019). Therapeutic efficacy of nanoparticles and routes of administration. Biomater. Res..

[168] Mclaughlin S., Podrebarac J., Ruel M., Suuronen E.J., McNeill B., Alarcon E.I. (2016). Nano-engineered biomaterials for tissue regeneration: What has been achieved so far?. Front. Mater..

[169] Soares S., Sousa J., Pais A., Vitorino C. (2018). Nanomedicine: Principles, Properties, and Regulatory Issues. Front. Chem..

[170] Su H., Wang Y., Gu Y., Bowman L., Zhao J., Ding M. (2018). Potential applications and human biosafety of nanomaterials used in nanomedicine. J. Appl. Toxicol..

[171] Zielińska A., Costa B., Ferreira M.V., Miguéis D., Louros JM S., Durazzo A., Lucarini M., Eder P., Chaud M.V., Morsink M. (2020). Nanotoxicology and Nanosafety: Safety-By-Design and Testing at a Glance. Int. J. Environ. Res. Public Health.

[172] Perez-Puyana V., Jiménez-Rosado M., Romero A., Guerrero A. (2020). Polymer-based scaffolds for soft-tissue engineering. Polymers.

[173] Garg T., Bilandi A., Kapoor B., Kumar S., Joshi R. (2011). Scaffold: Tissue engineering and regenerative medicine. Int. Res. J. Pharm..

[174] Reddy M.S.B., Ponnamma D., Choudhary R., Sadasivuni K.K. (2021). A Comparative Review of Natural and Synthetic Biopolymer Composite Scaffolds. Polymers.

[175] Donnaloja F., Jacchetti E., Soncini M., Raimondi M.T. (2020). Natural and Synthetic Polymers for Bone Scaffolds Optimization. Polymers.

